# Stakeholder perspectives on workplace-based performance assessment: towards a better understanding of assessor behaviour

**DOI:** 10.1007/s10459-017-9760-7

**Published:** 2017-02-02

**Authors:** Laury P. J. W. M. de Jonge, Angelique A. Timmerman, Marjan J. B. Govaerts, Jean W. M. Muris, Arno M. M. Muijtjens, Anneke W. M. Kramer, Cees P. M. van der Vleuten

**Affiliations:** 1 Department of Family Medicine, FHML, Maastricht University, P.O. Box 616, 6200 MD Maastricht, The Netherlands; 2 Department of Educational Research and Development, FHML, Maastricht University, Maastricht, The Netherlands; 3 Department of Family Medicine, Leiden University, Leiden, The Netherlands

**Keywords:** Assessment perceptions, Assessor variability, Competency based medical education, Q methodology, Workplace-based assessment

## Abstract

Workplace-Based Assessment (WBA) plays a pivotal role in present-day competency-based medical curricula. Validity in WBA mainly depends on how stakeholders (e.g. clinical supervisors and learners) use the assessments—rather than on the intrinsic qualities of instruments and methods. Current research on assessment in clinical contexts seems to imply that variable behaviours during performance assessment of both assessors and learners may well reflect their respective beliefs and perspectives towards WBA. We therefore performed a Q methodological study to explore perspectives underlying stakeholders’ behaviours in WBA in a postgraduate medical training program. Five different perspectives on performance assessment were extracted: Agency, Mutuality, Objectivity, Adaptivity and Accountability. These perspectives reflect both differences and similarities in stakeholder perceptions and preferences regarding the utility of WBA. In comparing and contrasting the various perspectives, we identified two key areas of disagreement, specifically ‘the locus of regulation of learning’ (i.e., self-regulated versus externally regulated learning) and ‘the extent to which assessment should be standardised’ (i.e., tailored versus standardised assessment). Differing perspectives may variously affect stakeholders’ acceptance, use—and, consequently, the effectiveness—of assessment programmes. Continuous interaction between all stakeholders is essential to monitor, adapt and improve assessment practices and to stimulate the development of a shared mental model. Better understanding of underlying stakeholder perspectives could be an important step in bridging the gap between psychometric and socio-constructivist approaches in WBA.

## Introduction

Workplace-Based Assessment (WBA) plays a pivotal role in present-day competency-based medical curricula. WBA essentially fulfils two functions: it serves a summative purpose, to enable decisions on the learner’s achievement, as well as a formative purpose, in order to drive learning and monitor personal development (van der Vleuten et al. 2010). The authenticity of the clinical environment implies that WBA is complex and typically influenced by uncontrolled variables such as case difficulty, patient mix and numbers. Moreover, validity in WBA mainly depends on how stakeholders (e.g. clinical supervisors, learners, programme directors) use the assessments—rather than on the intrinsic qualities of instruments and methods used to evaluate clinical performance (van der Vleuten and Verhoeven 2013). As a consequence, the utility of WBA is questioned regularly due to issues related to stakeholders’ behaviours in the assessment process.

Attempts to enhance the utility of WBA obviously target the quality of assessor judgements. There is a plethora of literature on assessor variability—and implications for WBA validity. Research findings reveal that assessor behaviours are quite persistent despite training and the idiosyncratic nature of assessor judgements may result in large differences between performance ratings (Cook et al. 2009; Govaerts et al. 2007, 2013; Holmboe et al. 2004). More specifically, findings indicate that a broad range of factors may underlie assessor variability, including cognitive factors (e.g. differences in leniency and stringency, stereotyping, categorisation) and the peculiarities of the (social or organisational) assessment context (Cook et al. 2010; Gawronski et al. 2003; Gingerich et al. 2011; Harasym et al. 2008; McManus et al. 2006; Sackett et al. 2002; Yeates et al. 2013). Within the current psychometric discourse in medical education, assessor variability is often seen as unwanted bias or error and assessment practices typically strive to objectify performance and to reach generalisable, reproducible judgements. However, it is increasingly being argued that there can be no such thing as ‘objective’ evaluation of performance (Newell and Shanks 2014). Taking a constructivist, sociocultural point of view, Govaerts et al. (2007), for instance, propose that assessors should be seen as active and goal-directed information processors, rather than passive measurement instruments (Govaerts et al. 2007).

The complexity of assessor judgements in WBA is clearly reflected in a model by Kogan et al. (2011), presenting multiple factors that explain the variability in judgements of trainee performance (Kogan et al. 2011). According to this model, assessors are not only driven by different frames of reference when observing and rating performance, they also use variable and therefore capricious approaches to translate judgements into numerical ratings. Moreover, assessors interpret trainee behaviour—for example exuding confidence or specific body language- and consequently make subjective inferences and assumptions about trainee performance. The model further suggests that assessment outcomes are also influenced by external factors, such as the clinical context of the observed encounter, the assessor-learner relationship and the (expected) response to- and acceptability of- feedback of both assessor and learner. Finally, there is an acknowledged role of the broader institutional culture in guiding assessor ratings; assessors’ beliefs about—and trust in—the assessment system seem to be crucial elements in the utility of assessment systems (Kogan et al. 2011). Performance assessment in workplace settings, then, is to be considered a ‘socially situated interpretive act’, in which a broad range of social and cognitive factors interact to produce idiosyncratic individual judgements of performance (Shay 2004). This latter postulate was reinforced by Berendonk et al. (2013), who pointed to the importance of assessors’ perspectives on assessment tasks and how these perspectives may influence assessment outcomes. More specifically, their study indicated that assessor behaviours are determined by (various) beliefs about assessment purposes and the utility of assessment for learning. These findings in medical education are consistent with those from research in industrial and organisational psychology indicating that performance ratings may be distorted by beliefs and perspectives that assessors have about the process of performance appraisal (Tziner et al. 1998, 2001, 2005).

Similarly, learners’ acceptance of work-based assessments and their use of feedback for competency development is not self-evident. In fact, a large body of research strongly suggests that learners’ beliefs about learning and learning outcomes filter and may even distort the message that feedback is intended to convey (Butler and Winne 1995). As such, different beliefs about the goals and meaning of feedback following performance evaluations in real-life workplace settings may impair learners’ acceptance and use of feedback (Embo et al. 2010). Teunissen et al. (2013) described the role of self-theories and their associated goal orientations in understanding the motivation underlying learners’ feedback-seeking behaviours—and thus the stance learners may adopt in WBA (Teunissen and Bok 2013): For example learners who are oriented towards learning goals regard feedback as useful information that helps to correct errors and achieve mastery; Performance-oriented learners, by contrast, tend to take feedback as a judgement about the self and as an indicator of inadequate ability. Especially when this judgement is perceived as negative, the conception of relevance and usage of feedback can be impaired. Finally, learners’ beliefs and attitudes towards fairness of assessments have been demonstrated to have an effect on the acceptance of feedback (Watling and Lingard 2012).

Current research on assessment in clinical contexts thus seems to imply that differing behaviours of both assessors and learners may well reflect their respective beliefs, perspectives and attitudes about WBA. This is well in line with theoretical frameworks that explain strong relationships between a person’s beliefs and intentions influencing actual behaviours, such as Ajzen and Madden’s Theory of Planned Behaviour (Ajzen 2002; Ajzen and Madden 1986). As a consequence, effective improvement of WBA may first and foremost require better understanding of stakeholders’ beliefs and perspectives. In the medical education realm, however, perspectives underlying behaviours in work-based assessment have received scant attention. The purpose of the present study is therefore to explore perspectives underlying stakeholders’ behaviours in WBA. Awareness of and knowledge on the content of underlying stakeholder perspectives may help us further enhance the utility and quality of performance assessment in competency-based medical education (CBME). To identify and describe key stakeholders’ perspectives regarding performance assessment in workplace settings, we used Q methodology, a well-known method for the systematic investigation of people’s viewpoints, beliefs and opinions regarding a certain topic(Watts and Stenner 2012).

## Method

### Context

This study was conducted at two General Practice (GP) Specialty Training institutes in the Netherlands. These GP programmes have an extensive track record in direct observation and feedback as cornerstones of competency-based education and assessment. The 3-year postgraduate training programme in the Netherlands consists of 2 years of training in general practice (years 1 and 3) and 1 year (year 2) of rotations in hospitals, mental health institutes and institutions for care of the elderly. Trainees spend 4 days in general practice and return to the training institute for a 1-day release programme every week. Throughout the training programme, a variety of formative and summative assessment methods are used periodically across all levels of Miller’s pyramid to evaluate the competency development of trainees. At the workplace, single encounter assessments (e.g. mini CEX, direct observations, case based discussions) are used for daily feedback and as input for an aggregated assessment portfolio based on the CanMEDS framework. This instrument is used as input for comprehensive competence assessment by supervisor, GP trainer and a psychologist teacher.

### Methodology

We performed this study using Q methodology, which fits well with our purpose of identifying and clarifying salient similarities and differences between various perspectives on WBA among stakeholders (Brown 2009; Cross 2005). Q methodology combines aspects of qualitative and quantitative research approaches and has successfully been applied in studies in health services (Harvey et al. 2013; Honey et al. 2013; Shabila et al. 2014; Stenner et al. 2003) and medical education (Fokkema et al. 2014; Ha 2014; Meade et al. 2013; Wallenburg et al. 2010). As described in the next paragraphs, Q methodology comprises four stages: (1) definition of the concourse surrounding a certain topic (i.e., WBA) and development of a statement set (Q set) based on the concourse; (2) identification of participants (P set); (3) ranking of statements (Q sort) by participants; and (4) statistical factor analysis, resulting in correlated clusters of Q sorts. These clusters can be interpreted as differing perspectives on the concourse—in our case differing stakeholder perspectives on performance assessment which will be described in the results section of this article(Van Exel 2005).

#### Concourse definition and development of the Q set

In Q methodology, the flow of communicability surrounding any topic is referred to as a ‘concourse’, and it is from this concourse that a sample of statements, the Q set, is subsequently drawn to enter a Q sort (Watts and Stenner 2012). In order to ensure coverage of all the relevant ground we developed our Q set through a conceptual review of the recent literature on work-based performance assessment in CBME. Key themes in the literature were identified and discussed iteratively within the research team. In addition, interviews were held with two experts with an extensive international track record in medical education research and two heads of a GP training institute in the Netherlands as a cross-check for the appropriate identification of key themes. Based on the conceptual literature review and interviews, we were able to identify three *key themes* in the concourse: ‘psychometric versus social constructivist approaches’, ‘holistic versus analytic conceptualisations of competence’ and ‘assessment *for* learning versus assessment *of* learning’.

The main researcher (LJ) formulated statements to represent the three themes, producing an initial set of 72 statements. LJ, AT and MG subsequently commented on the ambiguity, clarity and suitability of the statements in an iterative process, resulting in a pilot set of 52 statements. This Q set was then pilot-tested by three research team members (AT, JM, AK) and four potential participants (one GP teacher, two GP supervisors, one GP trainee), who were asked to complete a Q sort and comment on the completeness of the statement set, overlap and the applicability of statements. This resulted in a final set of 48 statements, which was approved by the research team. Finally, the statements were randomly numbered and printed on laminated cards. The complete list of statements used in the Q sort is depicted in Table 1.

**Table 1:** Complete list of 48 Q sort statements and idealised Q sorts for the five factors representing stakeholders’ perspectives on performance assessment in GP specialty training

No.	# Statement	Factor: perspectives				
		1: Agency	2: Mutuality	3: Objectivity	4: Adaptivity	5: Accountability
1.	Assessment of competency development in General Practice may only take place in the workplace setting	–5	−2	−2	−5	1^a^
2.	Acceptance of negative feedback necessitates a relationship of trust between trainee and supervisor	−2	+2	−3	+1	−2
3.	An assessment instrument should allow monitoring of trainee development	+3	+2	+2	+2	+1
4.	Giving feedback is important	+5	+5	+3	+5	+2
5.	In summative assessment, numerical grades are more appropriate than narrative evaluations	−2	−3	−1	0	−3
6.	For high−quality assessment, my experience as an assessor is more important than my experience as a (trainee or) general practitioner	−1	−1	+1	0	−1
7.	As an assessor, I feel appreciated by the training institute	0	–2^a^	+1	+3	+4
8.	Learners should be able to compensate for poor grades over time	−2	+2^a^	−3	−3	−4
9.	Assessment practices ensure high−quality patient care by the trainee	+2^a^	–3^a^	0	0	+5^a^
10.	Assessment should be based on the trainees’ learning goals and, consequently, be tailored to the individual trainee	0	+3^a^	–3	−1	−1
11.	Knowing whether an assessment is formative or summative is important	0	−1	+3	+2	−1
12.	It is important for a trainee to ask feedback	+4^b^	+2	0	0	+1
13.	Summative assessments cannot be conducted by the supervisor	−5	0	−2	−2	−3
14.	Assessment should primarily drive trainees’ learning process	+4^a^	0^b^	+2^a^	−2	−1
15.	Competencies cannot be evaluated with (numerical) grades	−4	0	0	−4	0
16.	A constructive cooperation between supervisor and trainee interferes with critical assessment practices	−3	−5	0^a^	−2	−4
17.	Assessment becomes more accurate due to the longitudinal relationship between supervisor and trainee	+1	+4	−1	+4	0
18.	Professional tasks are more easily entrusted to a trainee whose range of ideas and practices are similar to those of the GP supervisor	+2	+1	−1^b^	−4^b^	0
19.	Assessment interferes with the relationship between supervisor and trainee	−3	−4	−5	−3	−5
20.	A capable trainee is easy to recognise	+1	+1	−2^a^	+1	+1
21.	A trainee who performed well before may be expected to perform well again	+2	0	0	+1	+2
22.	I am a proficient assessor	+3	+1	+2	+3	+4
23.	Clear and precise assessment criteria are needed to assess a trainee accurately	+2^b^	−1	+4^b^	−1	0
24.	Numerical grades are not suitable for formative assessments	−2	−2	+3^a^	−2	−1
25.	When conducting an assessment, progressive development is more important than actual performance	−1	+3^a^	0	−3	−2
26.	An experienced supervisor is capable of conducting more accurate assessments	0	+2	+1	+3	+3
27.	As an assessor I feel involved with the training institute	0^a^	−5^a^	+1	+2	+2
28.	Assessment implies an additional workload	−1	−1	+2	0	+2
29.	If the purpose of the assessment is summative, my evaluations are stricter	−1	−2	−3	−1	0^b^
30.	Numerical grades allow me to assess accurately	−1	−3	−2	−1	−3
31.	Assessment within GP specialty training contributes to the future quality of general practitioners^c^	+5	+3	+5	+3	+3
32.	Assessment practices stimulate the competency development of trainees	+3	0	+4	0	+4
33.	It is important that a trainee and GP supervisor have shared perspectives on the GP profession	−4	−1	−4	−5	−1
34.	A trainee’s perspectives on the profession of general practice affect his/her assessment	+1	−4	−1	1	−3
35.	Assessors should judge in an identical fashion	0	+4	+2	0	−1
36.	Competencies are not to be assessed independently of one another	−3	0	−2	−2	−5^b^
37.	Summative assessments are more important than formative assessments	−4	−2	−4	−1	−2
38.	Professional tasks can be entrusted earlier to a trainee who self-directs his or her learning process	+4	0	0	+2	−2
39.	For the progressive development of competencies a trainee’s learning goals are more important than formal assessment criteria	−1	+3	0	0	+3
40.	When assessing a trainee, it is crucial that a trainee can perform professional tasks independently	0	−4	−1	−4	0
41.	When assessing a trainee, clear and precise assessment criteria are more important than the personal opinion of the supervisor	−3	−3	−1	−1	−4
42.	Rigorous assessment requires that both trainee and supervisor can receive feedback	+3	+5	+3	1^a^	+5
43.	Trainees are more likely to learn from narrative assessments than from numerical grades	+2	+4	+4	+4	+1
44.	In summative decisions previous formative assessments should not 45.be taken into consideration	−2	0	+1^b^	−2	−2
45.	Previous experiences with this trainee influence my assessment	+1	+1	−4^a^	+5^b^	+2
46.	My style of giving feedback is influenced by the way I expect it to be received	0	−1	−5^a^	+4^a^	0
47.	In the assessment process I include assessments of other assessors^c^	+1	+1	+1	+1	+3
48.	It is important to document assessments regularly	+1	+1	+5^b^	+2	+1

^a^Distinguishing statement (*P* < .01)

^b^Distinguishing statement (*P* < .05)

^c^Consensus statements (those that do not distinguish between ANY pair of factors, non-significant at *P* > .01)

#### Purposive selection of the P set

In Q methodology, participants must represent a broad range of expertise, roles and responsibilities related to the topic under study and specifically be able to express a defined viewpoint about (in our case) work-based performance assessment in CBME. In the Netherlands, the assessment of professional competence in general practice (GP) specialty training involves various stakeholders: a GP supervisor assesses the trainee through day-to-day observations; GP and psychologist teachers observe and provide feedback on different competences during the weekly 1-day release programme; The trainee is actively involved in the assessment process through regular self and peer assessments of competency development; while the programme director is responsible for high-stakes summative decisions based on the trainee’s portfolio. Hence, to cover all of the said stakeholder groups, we selected the P set through purposive stratified sampling from the various stakeholders involved in the assessment process. The P set consisted of 48 participants who were equally distributed between the two general practice specialty training institutes (See Table 2).

**Table 2:** P set representing stakeholders involved in performance assessment in CBME in two General Practice Specialty Training Institutes in the Netherlands

Training institute	Number of participants				
	GP supervisor (workplace)	GP teacher (STI^a^)	Psychologist teacher (STI^a^)	Programme director	GP trainee (1st year/3rd year)
Maastricht	10	3	2	1	4/4
Nijmegen	10	2	3	1	4/4

^a^General Practice Specialty Training Institute

#### Q-sorting procedure

**Fig. 1:**
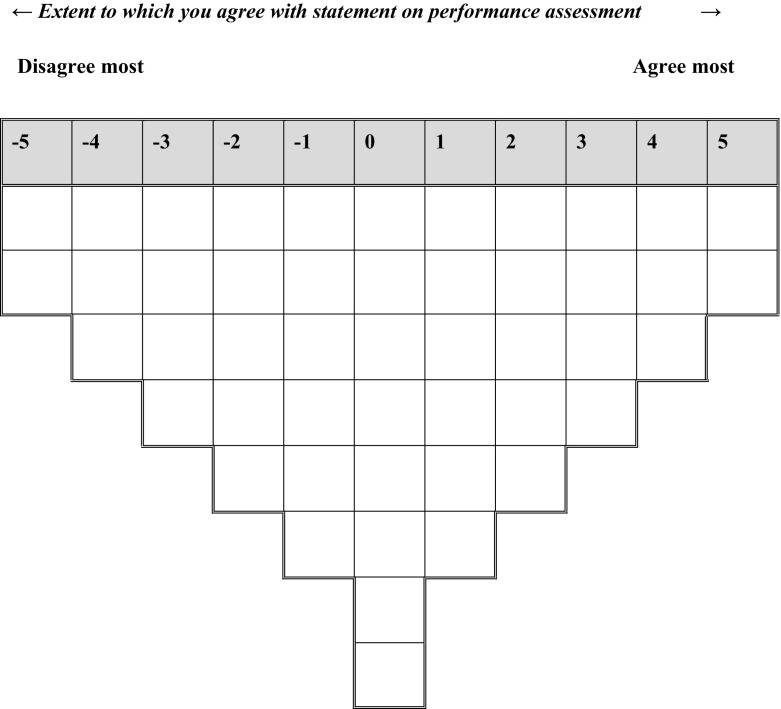
Sorting grid for the Q sort of 48 statements on work-based performance assessment in CBME

The purpose of the study and instructions for completing the Q-sorting task were described on an information sheet, which was handed out to each participant to obtain informed consent. We then asked participants to read through the statements of the Q set and start the Q-sorting procedure by dividing the statements into three piles: agree, disagree and neutral. A sorting grid with an 11-point distribution (−5 to +5) was used as a format to rank-order the laminated statement cards (Fig. 1). From the ‘disagree’ pile, participants were asked to select the two statements they disagreed with most and to place these in the two spaces at −5 (disagree most in the Q-sorting grid). After that, they returned to the disagree pile and continued sorting according to the Q-sorting grid until no statements were left from this pile. A similar process followed for the agree pile, after which the neutral pile was rank-ordered in the remaining middle area. A selection of participants provided verbal comments on the positioning of their Q-sorts which LJ collected immediately following the Q sort.

#### Statistical factor analysis

We analysed the data from the Q sorts using the PQMethod 2.35 programme (Schmolck 2014). All Q sorts were subjected to by-person factor analysis to uncover patterns in the rankings of statements, under the assumption that a high correlation between the Q sorts of certain participants indicated similarity of their viewpoints (Watts and Stenner 2012). The prominent common viewpoints were revealed in a three-step procedure. In a first step the scree plot corresponding to the Eigenvalues of the principal component analysis of the set of Q sorts was used to decide about the number of factors (common viewpoints) present in the data (Watts and Stenner 2005a). The corresponding number of components with highest eigenvalues were extracted as factors. Subsequently, the coordinate system of the factor space was rotated by varimax in order to optimize the loadings of the Q sorts for selection of subsets corresponding with a single factor. Only factors representing at least two Q sorts that exhibited a statistically significant correlation (*P* < .05) and having Eigenvalues greater than 1 were extracted (Shinebourne 2009). Here, the optimal number of factors was five. LJ, AT, MG and AM then examined the interpretability of the factor structures, which outcome confirmed that a five-factor solution indeed provided the most comprehensible fit.

As a next step, we created an idealised Q sort for each of the five factors (Table 1). These Q sorts indicated how a participant with that same perspective would have sorted the statements in the Q sorting grid (Fig. 1). Distinguishing statements (i.e., those statements that exceeded the difference score between any two factors at significance levels *P* < .05 or < .01) and consensus statements (i.e., those statements that did not distinguish between any pair of factors) were also identified (Table 1). For a holistic interpretation of the perspectives it is important to consider not only the extreme poles of the sorting but also and especially the relative positioning of the statements across the different factors. We therefore created arrays of differences representing the biggest differences in standard scores (Z scores) between any two factors (See "Appendix" section). All members of the research team iteratively interpreted and described each factor using the idealised Q sort, arrays of differences, distinguishing statements and comments provided by participants. Finally, to improve the validity of the interpretation of our results, we performed a brief member check with a representative sample of the participants. We asked 3 GP Supervisors, one GP trainee, one head of a GP training institute and one psychologist teacher of the GP training institute to comment on the viewpoint clusters.

## Ethical considerations

We performed this study between April 2014 and October 2014. It was approved by the Ethical Review Board of the Netherlands Association for Medical Education (NVMO-ERB; file number 313). Written informed consent was obtained from all participants, and the data were processed anonymously.

## Results

From the Q sorts of 48 participants, a five-factor solution emerged as the most comprehensible fit, representing five clearly distinguishable perspectives on work-based performance assessment in CBME. Each individual factor was significantly associated with three to nine participants, together accounting for 58% of total variance in the Q sorts. In the next paragraphs we will describe each factor (i.e., perspective), the corresponding number of the defining statements (e.g. #15) and their position in the idealised Q sort for that factor (e.g. +4). Each perspective will be clarified by means of an illustrative comment on the Q sort from one of the participants. For the complete list of numbered statements and their positions in the idealised Q sort, see Table 1. The table shows the pattern of level of agreement (on a scale −5 to +5) over all statements for each Factor, thereby defining the characteristics of that specific perspective. It should be emphasized that this table does not show loadings. In the Q factor analysis loadings are defined between participants and factors: participants with a score pattern over statements similar to the score pattern shown in one of the columns of the table show a high loading on that particular factor. The relation between the factor scores in a row is important: it expresses the between-factor differences in agreement for a statement. For each factor a selection of these score data for the subset of statements with a salient/significant score for that particular factor was used to reveal the characteristics of a perspective on WBA. The results of these analyses are presented below.

### Perspective 1: agency

This perspective holds that assessment should primarily guide the trainees’ learning process (#14: 4) and that feedback is central to learning. Active, self-directed learning on the part of the trainee plays a key role in the assessment process. It is especially important that the trainee actively seek feedback and that the assessor-supervisor provide this feedback (#4:+5; #12:+4). Summative assessments are not considered more important than formative assessments *(#*37:−4), connoting a commitment to assessment *for* learning and the learning process itself. It is essential that assessment instruments allow monitoring the development of trainees (#3:+3). A self-directed learning style implies that a trainee will ask for help when necessary, making supervisors feel comfortable to entrust professional tasks (#38:4). Both formative and summative performance assessments by the GP supervisor (#13:−5) support continuous assessment and guidance for learning, provided that clear and precise assessment criteria are available (#23:+2). Factor 1 explained 18% of variance and was defined by nine participants, five of which trainees, three GP supervisors and one GP trainer.

**Supervisor 9:** Most important is that trainees are able to learn, therefore, a test should primarily drive learning. The training institute should provide guidance, however, in the end it is the trainee who decides what is to be learnt. And that is fine with me: a capable trainee is able to do so.

### Perspective 2: mutuality

In this view, assessment should be embedded in the learning process and a joint responsibility of trainee and supervisor. To allow rigorous assessment, both trainee and supervisor must have the opportunity to receive feedback and trainees must be able to trust their supervisor (#42:+5; #2:+2). A constructive collaboration between supervisor and trainee is not perceived to interfere with critical assessment (#16:−5). In fact, when built on trust and used for learning, critical evaluation of performance can strengthen the trainee-supervisor relationship; assessment is more about process than it is about outcome (#19:−4). It is held, moreover, that the assessment becomes more accurate due to the longitudinal, mutual relationship between resident and supervisor (#17:+4). Continuity is also important in the assessment process itself: assessment should focus on progressive development (#25:+3) and learners should be able to compensate for poor grades over time (#8:+2). To guide trainees’ competency development towards professional standards, feedback is important (#4:+5). Trainees’ learning goals are considered more important than formal assessment criteria; Therefore, feedback should be tailored to trainees’ needs and goals (#39:+3; #10:+3). It is believed that trainees are more likely to learn from narrative assessments than from numerical grades (#43:+4), even in summative assessments (#5:−3). Prioritising the GP supervisor-trainee relationship, assessors sharing this perspective felt the least involved with—and appreciated by—the GP training institute compared to the other perspectives (#27:−5; 7:−2). Factor 2 explained 9% of the study variance and was defined by five participants: three trainees and two GP supervisors.

**Supervisor 2**: As a supervisor is assigned to one trainee for one year, you also have the time to invest in your relationship. By doing so, you are able to gain a clear impression of the trainee’s performance, not only of his medical skills, but also of his personal acting. This information is valuable, because it is also useful in general practice: how do you perceive a patient in relation to his context. When the relationship is good, you should be confident that an assessment is fair and that it is meant to drive learning. This only strengthens your relationship.

### Perspective 3: objectivity

Holders of this perspective attributed two different, yet equally important roles to assessment in GP specialty training (#37:−4): a formative role [*for* learning (#14:2; #32:+4)) and a summative one (*of* learning, to ensure future high-quality care (#31:+5)]. For the purpose of accountability, regular documentation of assessments by using an audit trail or portfolio was considered highly important (#48:5), as was the role of assessor, which required experience (#6:+1) and included specific tasks involving an additional workload (#28:+2). Assessment should be based on clear and precise criteria (#23:4), be objective and not biased or influenced by previous experiences (#45:−4), expectations (#20:−2), assessment purposes (#29:−3) or interpersonal relationships (#17:−1). Moreover, it should not be tailored to individual learning goals (#10:−3). Feedback must be honest and complete, without exceptions (#46:−5), and may even be unsolicited to advance competency development towards (external) standards (#12:0). The GP supervisor-trainee relationship must not be affected by the assessment (#19:−5), nor is it considered important that the resident and GP supervisor share hold similar perspectives on the GP profession (#33:−4). Numerical grades are not suitable for formative assessment as trainees are likely to learn more from narrative feedback than from numerical grades (#23:+3; #43:+4). Factor 3 explained 12% of the study variance and was defined by five participants: three GP supervisors, one GP teacher and one trainee.

**Supervisor 7:** ‘It is not necessary that one holds similar perspectives on the profession; friction can be productive. Criteria are important; it must be clear what the expectations of the training institute are.’

### Perspective 4: adaptivity

This view on performance assessment envisages a more flexible role for the assessor with regard to ownership of the assessment process: The relationship between supervisor and trainee should not necessarily be close (as opposed to the mutuality perspective of factor 2) and little weight is attached to the sharing of ideas about the GP profession, even when entrusting a trainee with specific professional tasks (#33:−5; #18:−4). Although feedback is allocated a prominent place (#4:+5), for rigorous assessment it is not necessary that both trainee and supervisor can receive feedback, suggesting a focus on one-way feedback delivery rather than a feedback dialogue (#42:1). Yet, assessors adjust their styles to the way they expect the feedback will be received (#46:+4), which stands in stark contrast with perspective 3 (objectivity), as does the acknowledgement that previous experiences influence assessment (#45:+5). While trainees are likely to learn more from narrative assessments than from numerical grades (#43:+4), it is certainly possible to evaluate competencies with grades (#15:−4), as long as this is supported by—and explained in—the training programme.

Additionally, it is believed that assessment becomes more accurate as the supervisor-trainee relationship develops (#17:+4) and as the supervisor accumulates experience (#26:+3). The training institute also fulfils an important role in providing more standardised guidance of the assessment process: assessment of competency development does not have to take place in the clinical setting only (#1:−5) and should not primarily drive a trainees’ learning process (#14:−2). This latter view reflects a proclivity towards external regulation which contrasts starkly with perspective 1 (agency). The relatively high scores on involvement with—and appreciation by—the training institute (#27:+2; #7:+3) also illustrate the importance of the role of the GP specialty training institute. Factor 4 explained 9% of the study variance and was defined by three participants: two GP supervisors and one trainee.

**Third-year trainee 5:** ‘You don’t need to have similar ideas about the profession. (…) You can be a very different type of general practitioner’.

**Supervisor 5:** Giving feedback is important. To give feedback to the trainee, I have to rely on my opinions and experience; however, in the end it is the training institute that determines what to do and how you should operate. By doing so, you can keep a ‘clean’ relationship with your trainee. After all, you depend on each other for one year.

### Perspective 5: accountability

From this standpoint, assessment practices do not only stimulate the competency development of trainees (#32:+4), but also serve to ensure high-quality patient care (#9:+5). In comparison with the other perspectives, this view holds that competency development should only be assessed in the clinical setting (#1:+1) and assessors do not experience any difficulties with assessing competencies independently of one another (#36:−5). Supervisors consider themselves proficient assessors (#22:+4) and feel involved with—(#27:+2) and much appreciated by (#7:+4) the GP specialty training institute. They also require experience to improve the accuracy of assessments (#26:+3) and there is room for idiosyncratic manoeuvre: they do not have to judge in an identical fashion (#35:−1) and personal opinions can be more important than (clear and precise) assessment criteria (#41:−4). The assessor is accountable for assessment in a mutual constructive relationship, where both trainee and supervisor can receive feedback (#42:+5). Assessment does not seem to jeopardise the supervisor-trainee relationship (#16:−4), not even in critical assessment practices (#19:−5). Moreover, assessors are demanding in the sense that they expect trainees to show a consistent level of performance over time, compensation over time is certainly not desirable (#8: −4). Finally, low priority is given to monitoring (#3:+1), indicating that trainees should perform well on each occasion. Factor 5 explained 10% of the study variance and was defined by three participants: two trainees and one GP supervisor.

**First-year trainee 1**: ‘The two of you have to go for it, otherwise it will not work. A proficient assessor is demanding, and also self-demanding. (…) Therefore, as a trainee, you also need to aim high’.

## Discussion

**Fig. 2:**
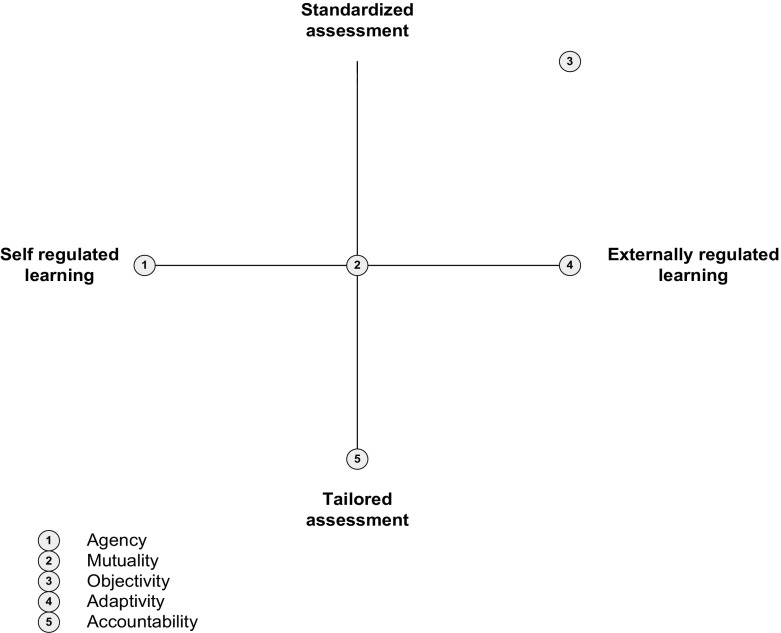
Conceptual space diagram depicting the positioning of the different perspectives on workplace-based assessment relative to the desired level of self-regulation/externally regulated learning and of standardisation/tailoring of assessment

In this study, we used Q methodology to identify and describe stakeholders’ perspectives on WBA in a postgraduate medical specialist training setting. We were able to extract five different perspectives on performance assessment: Agency, Mutuality, Objectivity, Adaptivity and Accountability. These perspectives reflect both differences and similarities in stakeholder perceptions and preferences regarding the use and utility of WBA. In comparing and contrasting the various perspectives, we identified two key areas of disagreement, specifically ‘the locus of regulation of learning’ (i.e., self-regulated versus externally regulated learning) and ‘the extent to which assessment should be standardised’ (i.e., tailored versus standardised assessment). Q methodology often uses a conceptual space diagram as a graphical representation of the different preferences and relationships between the various factors (perspectives) and as a means of identification of the pertinent disagreement areas (Milcu et al. 2014; Stenner et al. 2000; Watts and Stenner 2005a, b). By positioning the various perspectives and viewpoints relative to the axes representing areas of disagreement, possibilities for comparison and contrast across perspectives can be maximized (see Fig. 2). Whereas the horizontal axis reflects the desired level of self-regulated learning/externally regulated learning, the vertical axis refers to preferred levels of standardisation/tailoring of assessment. The various perspectives are positioned relative to these axes and to one another and are indicated by the numbers 1–5. In the following section, we will discuss the potential implications not only of differing, but also of shared perspectives among stakeholders for the utility of WBA in medical education practice.

The positions of the various perspectives along the horizontal axis in Fig. 2 clearly demonstrate that the importance attributed to self-regulation in learning and assessment differs widely amongst stakeholders. On the left-hand side of this axis, which point coincides with perspective 1 (Agency), stakeholders hold the view that trainees should actively self-direct their learning, take responsibility and show initiative in the assessment process. Halfway the axis, where perspective 2 (Mutuality) is located, the responsibility to identify learning needs through performance evaluations and feedback rests with both supervisor and trainee. Similarly, perspective 5 (Accountability) holds that supervisors must actively engage and take responsibility in the assessment process, and be driven by personal conceptions and beliefs about the role of assessment in ensuring high-quality patient care. At the other end of the spectrum we find both perspectives 3 (Objectivity) and 4 (Adaptivity) that perceive external criteria and requirements as main drivers of learning and WBA.

The differences in desired levels of self- and external regulation of learning in WBA, presented on the horizontal axis of our diagram, resonate with developments and research findings in medical education. Medical education institutions and accrediting regulatory bodies have acknowledged the importance of self-regulated learning for improving academic and clinical performance (Di Genova et al. 2015; Sandars and Cleary 2011). In the recently revised CanMEDS framework, there is a central role for self-regulated learning with regard to assessment: ‘a trainee has to develop, implement, monitor, and revise a personal learning plan to enhance professional practice’ (Frank et al. 2015). At the same time, the outcomes-based approach towards assessment in CBME almost inevitably implies the use of predefined and pre-specified competency frameworks. Detailed assessment criteria, performance standards and milestones may support learners and assessors in making decisions about achievement and inform future learning processes (ten Cate and Scheele 2007). In other words, by serving as a frame of reference, external assessment criteria may provide guidance and inform assessment decisions as well as the direction of future learning. Obviously, stakeholders’ perspectives on assessment, which translate into specific behaviours, will affect the utility of assessment systems.

Research findings strongly suggest that effective WBA implies a joint responsibility between learner and assessor (Holmboe et al. 2010; Norcini and Burch 2007). A prerequisite for effective self-regulated learning is that the supervisor entrusts at least part of the monitoring of learning goals to the learner and creates a learning environment that encourages mutual delivery and receipt of feedback (Pelgrim et al. 2012). In hierarchical learning environments, however, two-way feedback interaction is not self-evident; feedback often remains a supervisor-driven process, as reflected in perspective 5 (Accountability) (Archer 2010). When supervisors use personal conceptions of patient care or external assessment criteria, rather than personalised learning goals as the starting points for assessment activities, self-directed learning and engagement in self-monitoring of competency development can be impaired. On the other hand, excessive and exclusive reliance on learners’ initiatives and responsibility through self-assessments may also have a negative bearing on the utility of assessment processes. In fact, research findings consistently show that it is difficult to recognise one’s own incompetence, making self-assessment not the preferred mechanism to identify areas of personal weakness (Hodges et al. 2001; Regehr and Eva 2006). In this sense, external assessment frameworks can, indeed, serve an important purpose: to help identify learning needs and shortcomings learners have not been able to discover for themselves (Tochel et al. 2009). Yet, there is a caveat in that WBA may become a tick-box exercise if assessment is perceived to be completely driven by externally dictated criteria. Especially learners and assessors who strongly favour individualised assessment approaches will likely lack commitment and a sense of ownership in WBA.

Developers of assessment programmes must take into account the different user perspectives on WBA when combining summative and formative elements to stimulate assessment-for-learning. The effectiveness of assessments may depend on stakeholders’ beliefs and their associated perspectives on the assessment process. A lack of clarity regarding the purpose of the assessment, which is to promote self-regulated learning, may very well preclude successful implementation of WBA. In a study on learner-centeredness of a newly developed assessment programme, Bok et al. (2013), for instance, found inconsistencies between the learning function of assessment and its contribution to high-stakes decisions. Assessments that were designed as formative learning experiences were increasingly perceived as summative by learners, thereby undermining self-directed learning activities (Bok et al. 2013). In conclusion, mismatched, but also magnified shared perspectives on self- and externally regulated learning among stakeholders may result in serious impairment of the utility of work-based assessment practices.

The second area where stakeholders’ perceptions diverged concerns the preferred levels of standardisation of both assessment instruments and the assessment process itself. The perspectives presented on the vertical axis of the conceptual space diagram reflect these preferences. At the lower end of the vertical axis, perspective 5 (Accountability) holds that assessment must be based in the clinical context (also, contextualised) and tailored to the learning task at hand. Moreover, contextualised task requirements and related assessment criteria are regarded as more important than standardised and de-contextualised assessment criteria or performance standards. Expert judgement of clinical performance is considered crucial in ensuring that assessment is aligned with the requirements of high-quality patient care. Positioned in the middle range of the vertical axis, perspective 4 (Adaptivity) acknowledges the role of the social and organisational context of assessment. More specifically, feedback must be tailored to the characteristics of the clinical context and to the expectations of those involved in patient care. This emphasis on context specificity, however, is counterbalanced by programme developers and directors who are expected to guard the standardisation of the assessment process during medical training. Perspective 1 (Agency), too, recognises that trustworthy and fair high-stakes decision-making requires clear and predefined assessment criteria. At the same time, it holds that both formative and summative assessments should be tailored to the individual learning process. In between perspectives 1 and 4, we find perspective 2 (Mutuality) which, prioritising progressive development over time, prefers that assessment criteria be tailored to the learner’s needs and goals. To ensure that the assessment is robust, from perspective 2 care must be taken that assessors arrive at judgements in an identical, reproducible fashion. This focus on the accuracy and validity of assessments—psychometric criteria of assessment quality—is even stronger at the end of the range: Perspective 3 (Objectivity) clearly strives for objectification and standardisation of a performance assessment. Assessors should judge in an identical fashion, and there is a need for predefined clear and precise assessment criteria. From this perspective, adaptation of assessment to personal learning goals of the learner is considered as the least important.

Tensions arising from these opposing views on the level of standardisation of assessment in WBA resonate well with existing assessment literature. To enhance the accuracy of performance ratings (true scores capturing real, ‘objectified’ performance) from a quantitative psychometric point of view, the use of uniform test conditions and calibrated assessors is promoted. However, also within the field of psychometrics there is an ongoing debate about appropriate use of assessment instruments and interpretation of assessment results, specifically related to acknowledging the importance of individual differences as well as context (Schoenherr and Hamstra 2016). This is in line with discussions about utility of assessment protocols that can be tailored to the learning task at hand versus those that are pre-structured, detailed and standardised (Eva and Hodges 2012; Schuwirth and Swanson 2013).

In theory, assessors can be allowed more or less latitude in their judgments regardless of whether assessment tasks are selected by trainees as self-regulatory learners or chosen externally. As a matter of practice, however, having standardized assessments by standardized judges implies a limited number of things that can be assessed. Conversely, allowing trainees to choose whatever they want to be assessed would largely preclude standardized assessment because of constraints in time and effort in developing assessments. Non-standardized assessments that are tailored to the individual learner, however, may be perceived as biased, invalid, even unfair and less reliable than competence assessments in standardised assessment settings (Hodges 2013). The different stakeholder preferences with regard to the level of standardisation are also reflected in the way stakeholders (assessors and learners) perceive the utility of assessment instruments. Murphy (2008), for instance, found that stakeholders had diverging perceptions of the usefulness and qualities—and thus acceptability—of the various assessment formats used in GP specialist training (Murphy et al. 2008). It might be hypothesised that assessors whose primary concern is to eliminate subjectivity in the assessment process and to reach objective standardised judgements tend to prefer extensive and detailed rating scales; Assessors favouring a contextualised and holistic judgement, by contrast, are more likely to appreciate assessment frameworks that allow for tailored, individualised judgements (Kogan et al. 2015). When multiple assessors collectively take assessment decisions, then, these may be based on different perspectives. Moreover, differing personal views on the validity of assessment data can impair the utility of such team decisions. It is therefore crucial that any divergent preferences be spelt out in the decision-making process. If preferences regarding the level of standardisation of assessment criteria are appropriately aligned, assessors can work towards a shared mental model of functions and goals of assessment, and implications for assessment design (e.g. instruments, criteria). In conclusion, awareness of both differing and shared perspectives on assessment practices can increase mutual stakeholder understanding and therefore the utility of the assessment process. Mismatched preferences as to the needed level of standardisation of assessment can be a source of incomprehension and potentially obstruct effective assessment practices.

## Strengths and limitations

This study based on Q methodology has several strengths. First, we drew the Q set from a wide, representative range of current concourses in the domain of WBA. Second, our participant group consisted of different key stakeholders, representing the full assessment process in a general practice specialty postgraduate training setting. Third, our holistic approach to the data, taking into consideration the relative positions of all statements, resulted in a comprehensive and nuanced set of perspectives on WBA (Watts and Stenner 2005a, 2012). Finally, we found a statistically significant correlation between all five perspectives and different stakeholders in WBA. These findings indicate that the variability in perspectives results not only from the role of the stakeholder but also from particular preferences among individual stakeholders. The importance of understanding stakeholders’ perspectives on WBA is underpinned by psychological theories linking beliefs and intentions to behaviours (Ajzen and Madden 1986; Cilliers et al. 2015). The various perspectives on workplace-based performance assessment we identified amongst stakeholders may equally translate into different assessor and learner behaviours—fundamental to WBA validity and therefore utility.

There are some limitations to our study. First, the perspectives we described are not to be seen as archetypes for the classification of stakeholders. Individual stakeholders are likely to recognise aspects of several perspectives as their own, and perhaps identify with one of the presented perspectives more than with others. This is substantiated by comments of participants during the member checking procedure [e.g. “What I am trying to say here is, that although I recognise myself most in the perspective of Mutuality, this does not mean, that elements from the other four perspectives are strange to me, in the contrary” (psychologist teacher), or “I feel most comfortable with perspective 5 Accountability. However I do recognise elements of the other four perspectives, but less outspoken” (GP trainee)]. In addition, the aim of Q methodology is to sample the range and diversity of views expressed and not to make claims about the percentage of stakeholders expressing them. (Cross 2005) As a corollary, the generalisability of perspectives to specific subgroups (e.g. supervisor, trainee) is limited (Watts and Stenner 2005a).

Similarly, the transferability of the present findings to other medical specialties and work-based learning settings may be restricted. Our study was confined to a medical specialty postgraduate training setting characterised by long-term one-to-one contacts between supervisor and learner. In contrast, hospital-based supervisors typically have short-term contacts with multiple trainees. Although in these circumstances becoming aware of one’s perspectives on WBA is equally important, the lack of prolonged contact in the learning process may hamper the development of shared perspectives, impairing the utility of WBA. Second, we drew the statement set from a wide, representative range of current concourses in the domain of WBA. However, there are substantive differences between the various forms that WBA takes. WBA is not to restricted to a single tool neither is it used to assess a single skill only. A practical consequence of this could be that during the sorting procedure participants occasionally had a specific assessment tool or skill in mind, instead of the larger and more general concourse on WBA. Therefore our statement set consisted of 48 well balanced statements on WBA, representing a variety of tools and purposes.

### Implications for practice and research

WBA plays a pivotal role in competency-based medical curricula. The various perspectives and resulting behavioural differences in WBA may well explain why the implementation of competency-based assessment has proved so arduous (Hawkins et al. 2015; Holmboe 2014). Differing perspectives may variously affect stakeholders’ acceptance—and, consequently, the effectiveness—of assessment programmes.

Stakeholders in assessment practices should discuss the potential implications of both differing and shared perspectives on the utility of WBA, to avoid illegitimate inferences and interpretations of assessment outcomes. Excessive and exclusive reliance on only one of the stakeholders preferences may have a negative bearing on the utility of assessment processes. Therefore, establishing latitude regarding what is assessed and how it is assessed should be a joint responsibility.

Holmboe (2011) identified training of faculty in both clinical and assessment competence as ‘the missing link in CBME’ (Holmboe et al. 2011). Recent qualitative research by Kogan et al. (2015), moreover, revealed that assessors perceived training to positively influence specific assessment skills and to provide them with an enriched, more granular assessment vocabulary (Kogan et al. 2015) At the same time, these authors identified four factors that inhibited or precluded the application of training merits: some assessors preferred holistic assessments to checklists; they felt unable to define competence despite training; they experienced difficulty in changing their approach to assessment; or they expressed concern that they would be the firsts (that is, a minority) to adopt the new approach in their institution. In a recent review on user perceptions of WBA, Massie et al. (2015) identified three principal shortcomings of current WBA implementation: lack of clarity as to the purpose of WBAs, inadequate training in the provision of quality feedback in WBA and time constraints (Massie and Ali 2015). Although these findings support the need for adequate training of both assessors and learners increased awareness of various underlying perspectives not only by trainers, but also by assessment programme developers, will enhance the sophistication and utility of the assessment process.

Assessor judgements that deviate from the majority interpretation may represent important variants of how the assessment task is perceived (Gingerich et al. 2014). Variation in performance interpretation should be taken into account to arrive at a more comprehensive and coherent picture of a learner’s competence (Gingerich et al. 2011). Stakeholders in the assessment process should therefore be encouraged not only to document their performance interpretations, but also to articulate underlying values and assumptions in order to enhance WBA validity (Govaerts and van der Vleuten 2013). In summary, continuous interaction between all stakeholders is essential to monitor, adapt and improve assessment practices and to stimulate the development of a shared mental model (van der Vleuten et al. 2014). Future work should focus on the relation between specific perspectives and cognitive processing of individual stakeholders in WBA and actual behavioural approaches in assessment. This study has been exploring perspectives on WBA and contained small groups of stakeholders. As we stated, by definition in Q methodology the generalisability of perspectives to specific subgroups (e.g. supervisor, trainee) is limited (Watts and Stenner 2005a). Therefore, future work is needed to elucidate differences between the various stakeholder groups. Continued research in this arena, especially in the form of field or action research, will more clearly delineate the practical consequences of differing stakeholder perspectives on the utility of WBA.

## Conclusion

This study may contribute to our knowledge in the emerging field of assessor and learner cognition. It may enhance our understanding of the factors inhibiting and facilitating stakeholders’ acceptance of assessment systems and their trust in them, as well as of the effectiveness of feedback processes in performance assessment. This study indicates that stakeholders may very well hold different perspectives on goals and functions of WBA, which, in turn, may induce different perceptions of the role and responsibilities of the assessor and learner, assessment purposes, assessment process, and finally, the intended assessment outcome. Awareness and knowledge of stakeholder perspectives may deepen our understanding of stakeholders’ behaviours and interactions in assessment systems. Potential tensions amongst stakeholders, ensuing from different perspectives on and beliefs about WBA, mirror areas of concord and discord between prominent research perspectives (Gingerich et al. 2014). Our findings emphasise the importance of researchers and practitioners integrating aspects of different perspectives into a shared view. Awareness and understanding of underlying stakeholder perspectives could be an important step in bridging the gap between psychometric and socio-constructivist approaches in WBA.

## Appendix: Descending array of differences

**Table:**

Factor 1	Descending array of differences^a^	
*Factor 2*	*1* > *2* *9. Assessment practices assure high quality patient care by the trainee.* 2.119 *23. Clear and* precise *assessment criteria are needed to assess a trainee accurately.* 1.748 *38. Professional tasks can be entrusted earlier to a trainee who self*-*directs his or her learning process.* 1.602 *14. Assessment should primarily drive trainees´ learning process.* 1.571 *27. As an assessor I feel involved with the training institute.* 1.436	*2* > *1* *2. Acceptance of negative feedback necessitates a relationship of trust between trainee and supervisor.* 1.854 *35. Assessors should judge in an identical fashion.* 1.680 *8. Learners should be able to compensate for poor grades over time.* 1.603 *25. When conducting an assessment, progressive development is more important than actual performance.* 1.500 *39. For the progressive development of competencies a trainees’ learning goals are more important than formal assessment criteria.* 1.446
*Factor 3*	*1* > *3* *46. My style of giving feedback is influenced by the way I expect it to be received.* 2.066 *45. Previous experiences with this trainee influence my assessment.* 1.971 *12. It is important for a trainee to ask feedback.* 1.734 *38. Professional tasks can be entrusted earlier to a trainee who self*-*directs his or her learning process.* 1.465 *10. Assessment should be based on the trainees’ learning goals and, consequently, be tailored to the individual trainee.* 1.153	*3* > *1* *15. Competencies cannot be evaluated with (numerical) grades* 1.728 *24. Numerical grades are not suitable for formative assessments.* 1.611 *28. Assessment implies an additional workload.* 1.349 *35. Assessors should judge in an identical fashion.* 1.278 *48. It is important to document assessments regularly.* 1.258
*Factor 4*	*1* > *4* *14. Assessment should primarily drive trainees´ learning process.* 2.495 *18. Professional tasks are more easily entrusted to a trainee whose range of ideas and practices are similar to those of the GP supervisor.* 1.941 *40. When assessing a trainee, it is crucial that a trainee can perform professional tasks independently.* 1.631 *23. Clear and precise assessment criteria are needed to assess a trainee accurately.* 1.345 *12. It is important for a trainee to ask feedback.* 1.299	*4* > *1* *2. Acceptance of negative feedback necessitates a relationship of trust between trainee and supervisor.* 1.581 *37. Summative assessments are more important than formative assessments.* 1.285 *17. Assessment becomes more accurate due to the longitudinal relationship between supervisor and trainee.* 1.284 *45. Previous experiences with this trainee influence my assessment.* 1.224 *46. My style of giving feedback is influenced by the way I expect it to be received.* 1.153
*Factor 5*	*1* > *5* *14. Assessment should primarily drive trainees´ learning process.* 2.346 *38. Professional tasks can be entrusted earlier to a trainee who self*-*directs his or her learning process.* 2.271 *34. A trainee’s perspectives on the profession of general practice affect his/her assessment.* 1.503 *12. It is important for a trainee to ask feedback.* 1.181 *23. Clear and precise assessment criteria are needed to assess a trainee accurately.* 1.029	*5* > *1* *1. Assessment of competency development in General Practice may only take place in the workplace setting.* 2.209 *15. Competencies cannot be evaluated with (numerical) grades.* 1.887 *39. For the progressive development of competencies a trainees’ learning goals are more important than formal assessment criteria.* 1.362 *26. An experienced supervisor is capable of conducting more accurate assessments.* 1.210 *9. Assessment practices assure high quality patient care by the trainee.* 1.157

Factor 2	Descending array of differences	
*Factor 1*	*2* > *1* *2. Acceptance of negative feedback necessitates a relationship of trust between trainee and supervisor.* 1.854 *35. Assessors should judge in an identical fashion.* 1.680 *8. Learners should be able to compensate for poor grades over time.* 1.603 *25. When conducting an assessment, progressive development is more important than actual performance.* 1.500 *39. For the progressive development of competencies a trainees’ learning goals are more important than formal assessment criteria.* 1.446	*1* > *2* *9. Assessment practices assure high quality patient care by the trainee* 2.119 *23. Clear and precise assessment criteria are needed to assess a trainee accurately.* 1.748 *38. Professional tasks can be entrusted earlier to a trainee who self*-*directs his or her learning process.* 1.602 *14. Assessment should primarily drive trainees´ learning process.* 1.571 *27. As an assessor I feel involved with the training institute.* 1.436
*Factor 3*	*2* > *3* *10. Assessment should be based on the trainees’ learning goals and, consequently, be tailored to the individual trainee.* 2.304 *45. Previous experiences with this trainee influence my assessment.* 2.163 *17. Assessment becomes more accurate due to the longitudinal relationship between supervisor and trainee.* 2.119 *2. Acceptance of negative feedback necessitates a relationship of trust between trainee and supervisor.* 1.887 *8. Learners should be able to compensate for poor grades over time.* 1.751	*3* > *2* *23. Clear and precise assessment criteria are needed to assess a trainee accurately.* 2.392 *27. As an assessor I feel involved with the training institute.* 2.212 *32. Assessment practices stimulate the competency development of trainees.* 1.803 *16. A constructive cooperation between GP supervisor and trainee interferes with critical assessment practices.* 1.784 *24. Numerical grades are not suitable for formative assessments.* 1.729
*Factor 4*	*2* > *4* *25. When conducting an assessment, progressive development is more important than actual performance.* 2.186 *18. Professional tasks are more easily entrusted to a trainee whose range of ideas and practices are similar to those of the GP supervisor.* 1.970 *42. Rigorous assessment requires that both trainee and supervisor can receive feedback.* 1.939 *8. Learners should be able to compensate for poor grades over time.* 1.844 *33. It is important that trainee and GP supervisor have shared perspectives on the GP profession.* 1.659	*4* > *2* *27. As an assessor I feel involved with the training institute.* 2.478 *46. My style of giving feedback is influenced by the way I expect it to be received.* 2.024 7. *As an assessor, I feel appreciated by the training institute*. 1.936 *34. A trainee’s perspectives on the profession of general practice affect his/her assessment.* 1.404 *11. Knowing whether an assessment is formative or summative is important.* 1.244
*Factor 5*	*2* > *5* *8. Learners should be able to compensate for poor grades over time.* 2.164 *25. When conducting an assessment, progressive development is more important than actual performance.* 1.844 *2. Acceptance of negative feedback necessitates a relationship of trust between trainee and supervisor.* 1.813 *10. Assessment should be based on the trainees’ learning goals and, consequently, be tailored to the individual trainee.* 1.689 *35. Assessors should judge in an identical fashion.* 1.659	*5* > *2* *9. Assessment practices assure high quality patient care by the trainee.* 3.276 *27. As an assessor I feel involved with the training institute.* 2.363 7. *As an assessor, I feel appreciated by the training institute*. 2.053 *32. Assessment practices stimulate the competency development of trainees.* 1.575 *28. Assessment implies an additional workload.* 1.380

Factor 3	Descending array of differences	
*Factor 1*	*3* > *1* *15. Competencies cannot be evaluated with (numerical) grades* 1.728 *24. Numerical grades are not suitable for formative assessments.* 1.611 *28. Assessment implies an additional workload.* 1.349 *35. Assessors should judge in an identical fashion.* 1.278 *48. It is important to document assessments regularly.* 1.258	*1* > *3* *46. My style of giving feedback is influenced by the way I expect it to be received.* 2.066 *45. Previous experiences with this trainee influence my assessment.* 1.971 *12. It is important for a trainee to ask feedback.* 1.734 *38. Professional tasks can be entrusted earlier to a trainee who self*-*directs his or her learning process.* 1.465 *10. Assessment should be based on the trainees’ learning goals and, consequently, be tailored to the individual trainee.* 1.153
*Factor 2*	*3* > *2* *23. Clear and precise assessment criteria are needed to assess a trainee accurately.* 2.392 *27. As an assessor I feel involved with the training institute.* 2.212 *32. Assessment practices stimulate the competency development of trainees.* 1.803 *16. A constructive cooperation between GP supervisor and trainee interferes with critical assessment practices.* 1.784 *24. Numerical grades are not suitable for formative assessments.* 1.729	*2* > *3* *10. Assessment should be based on the trainees’ learning goals and, consequently, be tailored to the individual trainee.* 2.304 *45. Previous experiences with this trainee influence my assessment.* 2.163 *17. Assessment becomes more accurate due to the longitudinal relationship between supervisor and trainee.* 2.119 *2. Acceptance of negative feedback necessitates a relationship of trust between trainee and supervisor.* 1.887 *8. Learners should be able to compensate for poor grades over time.* 1.751
*Factor 4*	*3* > *4* *23. Clear and precise assessment criteria are needed to assess a trainee accurately.* 1.989 *14. Assessment should primarily drive trainees´ learning process* 1.766 *24. Numerical grades are not suitable for formative assessments.* 1.723 *15. Competencies cannot be evaluated with (numerical) grades.* 1.435 *32. Assessment practices stimulate the competency development of trainees.* 1.347	*4* > *3* *46. My style of giving feedback is influenced by the way I expect it to be received.* 3.219 *45. Previous experiences with this trainee influence my assessment.* 3.195 *17. Assessment becomes more accurate due to the longitudinal relationship between supervisor and trainee.* 1.997 *2. Acceptance of negative feedback necessitates a relationship of trust between trainee and supervisor.* 1.614 *20. A capable trainee is easy to recognize.* 1.160
*Factor 5*	*3* > *5* *23. Clear and precise assessment criteria are needed to assess a trainee accurately.* 1.674 *14. Assessment should primarily drive trainees´ learning process.* 1.616 *16. A constructive cooperation between GP supervisor and trainee interferes with critical assessment practices.* 1.473 *35. Assessors should judge in an identical fashion.* 1.257 *48. It is important to document assessments regularly.* 1.200	*5* > *3* *45. Previous experiences with this trainee influence my assessment.* 2.416 *9. Assessment practices assure high quality patient care by the trainee.* 2.246 *46. My style of giving feedback is influenced by the way I expect it to be received.* 1.849 *1. Assessment of competency development in General Practice may only take place in the workplace setting.* 1.342 *39. For the progressive development of competencies a trainees’ learning goals are more important than formal assessment criteria.* 1.244

Factor 4	Descending array of differences	
*Factor 1*	*4* > *1* *2. Acceptance of negative feedback necessitates a relationship of trust between trainee and supervisor.* 1.581 *37. Summative assessments are more important than formative assessments.* 1.285 *17. Assessment becomes more accurate due to the longitudinal relationship between supervisor and trainee.* 1.284 *45. Previous experiences with this trainee influence my assessment.* 1.224 *46. My style of giving feedback is influenced by the way I expect it to be ask feedback.* 1.153	*1* > *4* *14. Assessment should primarily drive trainees´ learning process.* 2.495 *18. Professional tasks are more easily entrusted to a trainee whose range of ideas and practices are similar to those of the GP supervisor.* 1.941 *40. When assessing a trainee, it is crucial that a trainee can perform professional tasks independently.* 1.631 *23. Clear and precise assessment criteria are needed to assess a trainee accurately.* 1.345 *12. It is important for a trainee to ask feedback.* 1.299
*Factor 2*	*4* > *2* *27. As an assessor I feel involved with the training institute.* 2.478 *46. My style of giving feedback is influenced by the way I expect it to be received.* 2.024 7. *As an assessor, I feel appreciated by the training institute*. 1.936 *34. A trainee’s perspectives on the profession of general practice affect his/her assessment.* 1.404 *11. Knowing whether an assessment is formative or summative is important.* 1.244 2	*2* > *4* *25. When conducting an assessment, progressive development is more important than actual performance.* 2.186 *18. Professional tasks are more easily entrusted to a trainee whose range of ideas and practices are similar to those of the GP supervisor.* 1.970 *42. Rigorous assessment requires that both trainee and supervisor can receive feedback.* 1.939 *8. Learners should be able to compensate for poor grades over time.* 1.844 *33. It is important that trainee and GP supervisor have shared perspectives on the GP profession.* 1.659
*Factor 3*	*4* > *3* *46. My style of giving feedback is influenced by the way I expect it to be received.* 3.219 *45. Previous experiences with this trainee influence my assessment.* 3.195 *17. Assessment becomes more accurate due to the longitudinal relationship between supervisor and trainee.* 1.997 *2. Acceptance of negative feedback necessitates a relationship of trust between trainee and supervisor.*1.614 *20. A capable trainee is easy to recognize.* 1.160	*3* > *4* *23. Clear and precise assessment criteria are needed to assess a trainee accurately.* 1.989 *14. Assessment should primarily drive trainees´ learning process* 1.766 *24. Numerical grades are not suitable for formative assessments.* 1.723 *15. Competencies cannot be evaluated with (numerical) grades.* 1.435 *32. Assessment practices stimulate the competency development of trainees.*1.347
*Factor 5*	*4* > *5* *38. Professional tasks can be entrusted earlier to a trainee who self*-*directs his or her learning process.* 1.785 *2. Acceptance of negative feedback necessitates a relationship of trust between trainee and supervisor.* 1.540 *34. Perspectives of a trainee on the profession of General Practice affect his assessment.* 1.484 *46. My style of giving feedback is influenced by the way I expect it to be received.* 1.370 *17. Assessment becomes more accurate due to the longitudinal relationship between GP supervisor and trainee.* 1.308	*5* > *4* *1. Assessment of competency development in General Practice may only take place in the workplace setting.* 2.404 *9. Assessment practices assure high quality patient care by the trainee.* 2.277 *33. It is important that trainee and GP supervisor have shared perspectives on the GP profession.* 1.802 *42. Rigorous assessment requires that both trainee and supervisor can receive feedback.* 1.778 *40. When assessing a trainee, it is crucial that a trainee can perform professional tasks independently.* 1.600

Factor 5	Descending array of differences	
*Factor 1*	*5* > *1* *1. Assessment of competency development in General Practice may only take place in the workplace setting.* 2.209 *15. Competencies cannot be evaluated with (numerical) grades.* 1.887 *39. For the progressive development of competencies a trainees’ learning goals are more important than formal assessment criteria.* 1.362 *26. An experienced supervisor is capable of conducting more accurate assessments.* 1.210 *9. Assessment practices assure high quality patient care by the trainee.* 1.157	*1* > *5* *14. Assessment should primarily drive trainees´ learning process.* 2.346 *38. Professional tasks can be entrusted earlier to a trainee who self*-*directs his or her learning process.* 2.271 *34. A trainee’s perspectives on the profession of general practice affect his/her assessment.* 1.503 *12. It is important for a trainee to ask feedback.* 1.181 *23. Clear and precise assessment criteria are needed to assess a trainee accurately.* 1.029
*Factor 2*	*5* > *2* *9. Assessment practices assure high quality patient care by the trainee.* 3.276 *27. As an assessor I feel involved with the training institute.* 2.363 7. *As an assessor, I feel appreciated by the training institute*. 2.053 *32. Assessment practices stimulate the competency development of trainees.* 1.575 *28. Assessment implies an additional workload.* 1.380	*2* > *5* *8. Learners should be able to compensate for poor grades over time.* 2.164 *25. When conducting an assessment, progressive development is more important than actual performance.* 1.844 *2. Acceptance of negative feedback necessitates a relationship of trust between trainee and supervisor.* 1.813 *10. Assessment should be based on the trainees’ learning goals and, consequently, be tailored to the individual trainee.* 1.689 *35. Assessors should judge in an identical fashion.* 1.659
*Factor 3*	*5* > *3* *45. Previous experiences with this trainee influence my assessment.* 2.416 *9. Assessment practices assure high quality patient care by the trainee.* 2.246 *46. My style of giving feedback is influenced by the way I expect it to be received.* 1.849 *1. Assessment of competency development in General Practice may only take place in the workplace setting.* 1.342 *39. For the progressive development of competencies a trainees’ learning goals are more important than formal assessment criteria.* 1.244	*3* > *5* *23. Clear and precise assessment criteria are needed to assess a trainee accurately.* 1.674 *14. Assessment should primarily drive trainees´ learning process.* 1.616 *16. A constructive cooperation between GP supervisor and trainee interferes with critical assessment practices.* 1.473 *35. Assessors should judge in an identical fashion.* 1.257 *48. It is important to document assessments regularly.* 1.200
*Factor 4*	*5* > *4* *1. Assessment of competency development in General Practice may only take place in the workplace setting.* 2.404 *9. Assessment practices assure high quality patient care by the trainee.* 2.277 *33. It is important that trainee and GP supervisor have shared perspectives on the GP profession.* 1.802 *42. Rigorous assessment requires that both trainee and supervisor can receive feedback.* 1.778 *40. When assessing a trainee, it is crucial that a trainee can perform professional tasks independently.* 1.600	*4* > *5* *38. Professional tasks can be entrusted earlier to a trainee who self*-*directs his or her learning process.* 1.785 *2. Acceptance of negative feedback necessitates a relationship of trust between trainee and supervisor.* 1.540 *34. A trainee’s perspectives on the profession of general practice affect his/her assessment.* 1.484 *46. My style of giving feedback is influenced by the way I expect it to be received.* 1.370 *17. Assessment becomes more accurate due to the longitudinal relationship between supervisor and trainee.* 1.308

^a^The descending array of differences shows the differences between Z-scores of any pair of factors
